# Deep learning analysis to predict *EGFR* mutation status in lung adenocarcinoma manifesting as pure ground-glass opacity nodules on CT

**DOI:** 10.3389/fonc.2022.951575

**Published:** 2022-09-02

**Authors:** Hyun Jung Yoon, Jieun Choi, Eunjin Kim, Sang-Won Um, Noeul Kang, Wook Kim, Geena Kim, Hyunjin Park, Ho Yun Lee

**Affiliations:** ^1^ Department of Radiology and Center for Imaging Science, Samsung Medical Center, Sungkyunkwan University School of Medicine, Seoul, South Korea; ^2^ Department of Radiology, Veterans Health Service Medical Center, Seoul, South Korea; ^3^ Department of Artificial Intelligence, Sungkyunkwan University, Suwon, South Korea; ^4^ Department of Electrical and Computer Engineering, Sungkyunkwan University, Suwon, South Korea; ^5^ Division of Pulmonary and Critical Care Medicine, Department of Medicine, Samsung Medical Center, Sungkyunkwan University School of Medicine, Seoul, South Korea; ^6^ Department of Health Science and Technology, Samsung Advanced Institute for Health Sciences & Technology (SAIHST), Sungkyunkwan University, Seoul, South Korea; ^7^ Division of Allergy, Department of Medicine, Samsung Medical Center, Sungkyunkwan University School of Medicine, Seoul, South Korea; ^8^ Center for Neuroscience Imaging Research, Institute for Basic Science, Suwon, South Korea; ^9^ School of Electronic and Electrical Engineering, Sungkyunkwan University, Suwon, South Korea

**Keywords:** lung adenocarcinoma, ground-glass opacity nodule, computed tomography, deep learning, epidermal growth factor receptor

## Abstract

**Background:**

Epidermal growth factor receptor-tyrosine kinase inhibitors (*EGFR*-TKIs) showed potency as a non-invasive therapeutic approach in pure ground-glass opacity nodule (pGGN) lung adenocarcinoma. However, optimal methods of extracting information about *EGFR* mutation from pGGN lung adenocarcinoma images remain uncertain. We aimed to develop, validate, and evaluate the clinical utility of a deep learning model for predicting *EGFR* mutation status in lung adenocarcinoma manifesting as pGGN on computed tomography (CT).

**Methods:**

We included 185 resected pGGN lung adenocarcinomas in the primary cohort. The patients were divided into training (n = 125), validation (n = 23), and test sets (n = 37). A preoperative CT-based deep learning model with clinical factors as well as clinical and radiomics models was constructed and applied to the test set. We evaluated the clinical utility of the deep learning model by applying it to 83 GGNs that received *EGFR*-TKI from an independent cohort (clinical validation set), and treatment response was regarded as the reference standard.

**Results:**

The prediction efficiencies of each model were compared in terms of area under the curve (AUC). Among the 185 pGGN lung adenocarcinomas, 122 (65.9%) were *EGFR*-mutant and 63 (34.1%) were *EGFR*-wild type. The AUC of the clinical, radiomics, and deep learning with clinical models to predict *EGFR* mutations were 0.50, 0.64, and 0.85, respectively, for the test set. The AUC of deep learning with the clinical model in the validation set was 0.72.

**Conclusions:**

Deep learning approach of CT images combined with clinical factors can predict *EGFR* mutations in patients with lung adenocarcinomas manifesting as pGGN, and its clinical utility was demonstrated in a real-world sample.

## Introduction

Detection of epidermal growth factor receptor (*EGFR*) mutations for lung adenocarcinoma is crucial since tyrosine kinase inhibitors (TKI) are tailored for treatments in lung adenocarcinoma with *EGFR* mutations (1–3). Approximately 80% of patients with *EGFR*‐mutant lung cancer respond to *EGFR-* TKIs therapy at initial treatment (4).

Due to the growing clinical use of low-dose computed tomography (CT) screening for lung cancer (5–7), pulmonary pure ground-glass opacity nodules (pGGN) are becoming clinically important in oncology especially for management given its diagnosis in practice is increasing, and the incidence of cancer in pGGN may be as high as 63% (8). In addition, around 20–30% of resected GGN were accompanied by multiple synchronous pGGNs (9), and there have been reports of developing metachronous pGGNs with an incidence of 2% after surgery in primary lung cancer (10). Therefore, there is a dilemma regarding how to deal with synchronous and metachronous pGGNs. Moreover, surgical therapy for pGGNs may be unfeasible for patients with poor pulmonary function or when lesions have central locations that make it difficult to perform repeated limited resection (11).

In terms of such a challenging condition, a few reports have shown the potency of molecular targeted therapy, *EGFR*-TKI as a novel strategy for the treatment of cases with multiple GGNs, and they helped provide a non-invasive therapeutic approach for *EGFR*-mutated lung adenocarcinoma manifesting as pGGN (12, 13). The authors used surgical resection for the major lesion which was the most invasive, and continued *EGFR*-TKI gefitinib treatment for unresectable GGNs (more than 10mm), and they achieved a complete response (12, 13). Additionally, there can be difficulty accessing tissue samples of pGGN through core biopsy due to the potential risk of complications and limitations in pathologic evaluation such as stromal invasion (14, 15). With such clinical conditions, *EGFR* mutation prediction using a noninvasive method such as imaging of lung adenocarcinoma manifesting as pGGN is desirable.

Models for predicting *EGFR* mutations on imaging have been developed using a radiomics approach (16–18), but these methods only reflect generalized adenocarcinomas and lack specificity for pGGN. Radiomics also rely on precise tumor boundary annotation, which requires manual labeling, and interobserver reproducibility and robustness of results are relatively unsatisfactory (19–21). In contrast, advanced artificial intelligence models can overcome these problems through self-learning strategies such as deep learning (22, 23). Deep learning models have shown promising performance in assisting lung cancer analysis (24–27). Nevertheless, deep learning models for the prediction of *EGFR* mutation in lung adenocarcinoma manifesting as pGGN have not been evaluated thus far. Besides, development and validation of models to deal with pGGN in particular are complicated and difficult due to need for copious data collection and image processing. Therefore, extraction of *EGFR* mutation information from lung adenocarcinoma manifesting as pGGN on images remains uncertain. Furthermore, there have been no attempts to evaluate the clinical utility of deep learning models by performance validation through the testing of clinically meaningful endpoints (28, 29).

Thus, we developed and validated a CT-based deep learning model with clinical factors for predicting *EGFR* mutation status in lung adenocarcinoma manifesting as pGGN. We demonstrated its clinical utility using an independent data set of patients who received *EGFR*-TKI and evaluated treatment response as the reference standard.

## Methods

### Patients

**Figure 1:**
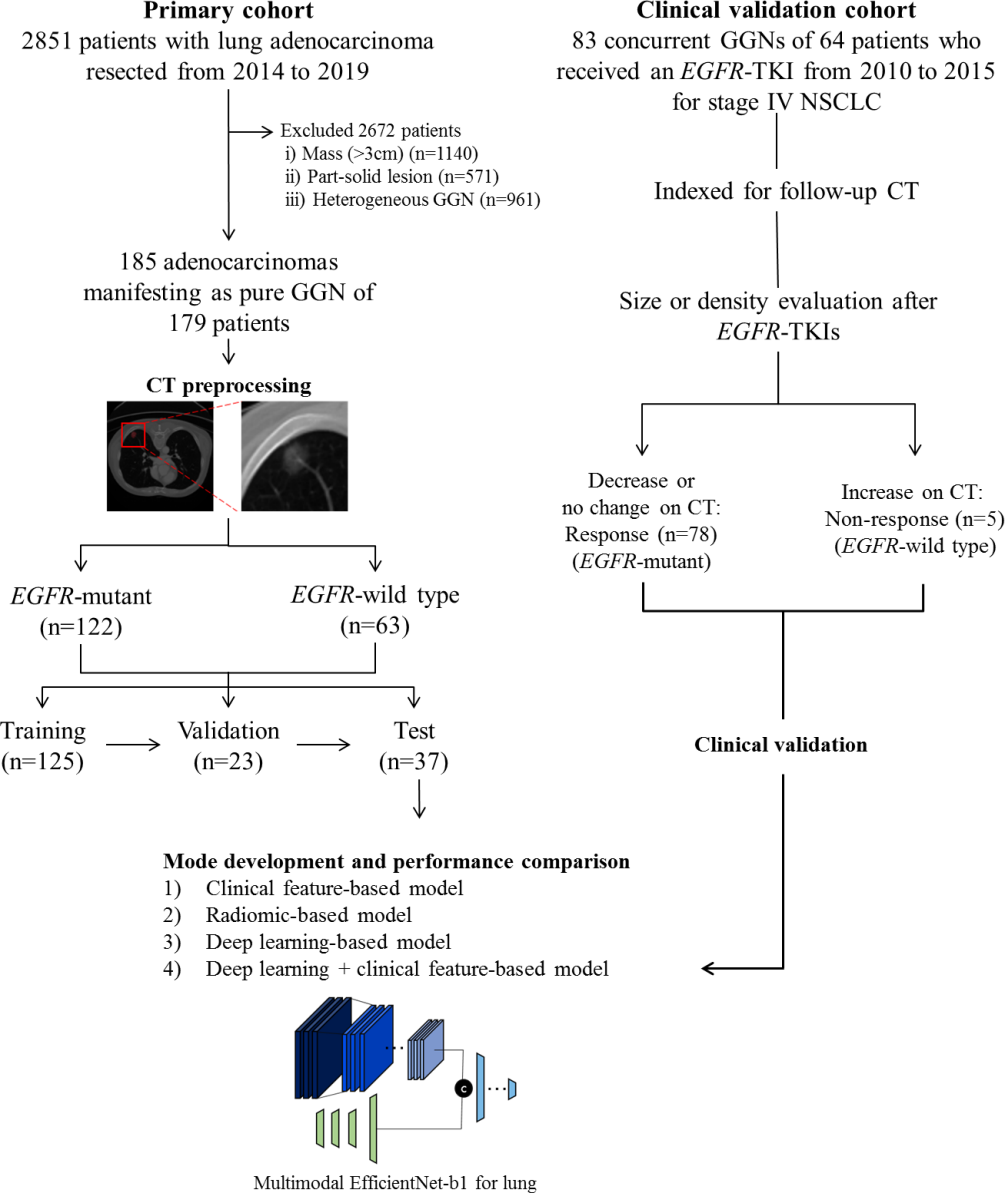
Flow diagram describing the development of the *EGFR* mutation prediction model in this study.

Our institutional review board approved this retrospective study, and the requirement for informed consent was waived. We conducted a retrospective chart review and identified 2,851 patients who had undergone surgical resection for lung adenocarcinoma as initial curative resection from January 2014 to August 2019. Patients who met the following inclusion criteria were included in this study: 1) histologically confirmed primary lung adenocarcinoma; 2) pathological examination of tumor specimens carried out with proven records of *EGFR* mutation status; 3) pre-operative chest CT data obtained; and 4) CT findings of the tumor showed pGGN. Patients were excluded if 1) clinical data including age, sex, and smoking history were missing; or 2) CT findings of the tumor showed a large mass (>3 cm), part-solid lesion, or heterogeneous GGN. Finally, 185 pGGN adenocarcinomas of 179 patients of were included in the primary cohort (model training [n = 125 nodules of 120 patients], technical validation [n = 23 of 23 patients], and tests [n = 37 of 36 patients]). We randomly divided cohorts into training, validation, and test sets maintaining the ratio of EGFR-mutant and EGFR wild type (**Figure 1**).

### Data collection and EGFR mutational profiling

Clinical data were collected from electronic medical records at the time of diagnostic workup. Sex, age, smoking status, Union for International Cancer Control stage, and operation type were recorded. Histologic reports were also retrieved from electronic medical records with histological classifications based on the International Association for the Study of Lung Cancer/American Thoracic Society/European Respiratory Society multidisciplinary classification of lung adenocarcinoma (30).

*EGFR* mutations for lung adenocarcinoma were identified using a PNA clamp kit or real-time polymerase chain reaction (31). Wild-type *EGF*R in this study referred to no mutations detected among those loci.

### CT image acquisition

Heterogeneity in the imaging acquisition protocols was inevitable, as data were obtained retrospectively at a tertiary referral center. All patients underwent CT scans from the lung apex to the base at suspended maximum inspiration. Scans were performed at 120 kVp with mAs ranging at 150–200 mAs and detector collimation was 1.25 or 0.625 mm. CT scans were reconstructed with slice thickness less than or equal to 2.5 mm. Slice increments were equal to or less than the slice thickness. All CT scans included axial reconstruction and most CT scans also had coronal reformatted images. Most patients underwent contrast-enhanced CT scans at a scan delay of 60 s after contrast material injection. All helical CT images were obtained using a high-quality 16 or 64-channel multidetector CT scanner.

### Data preprocessing

The tumor region of interest (ROI) was automatically segmented for all patients in each dataset using commercial software (Aview, version 1.0.23, 2018; Coreline Soft, Seoul, Korea) to generate a volume of interest that included the entire target lesion (32). Additional manual correction was performed to exclude bronchovascular structures and the borders of ground-glass opacities by a thoracic radiologist (HYL, 15 years of experience). Since CT imaging resolution varied within and across the two cohorts, isotropic resampling to 1 mm x 1mm x 1mm was conducted. Resampling was performed with b-spline interpolation for CT images and with the nearest neighbor method for ROI. For deep learning methods, the center of the tumor was calculated as the centroid of the ROI and then we extracted one center slice and two additional slices positioned 3 mm below and above the center slice in the axial direction. The three slices were combined as a three-channel image mimicking the color red/green/blue channels of the natural image. We then cropped each image around the center of the tumor to a size of 64 mm. Finally, all tumor regions were represented in 64×64×3 image patches. The intensities were normalized with min-max scaling. As the clinical variable, sex was binarized with values 0 for female and 1 for male. Age was normalized between 0 and 1 with min-max scaling. Smoking status was also binarized with 0 representing never smokers and 1 representing others.

### Multimodal EfficientNet-b1 for lung

**Figure 2:**
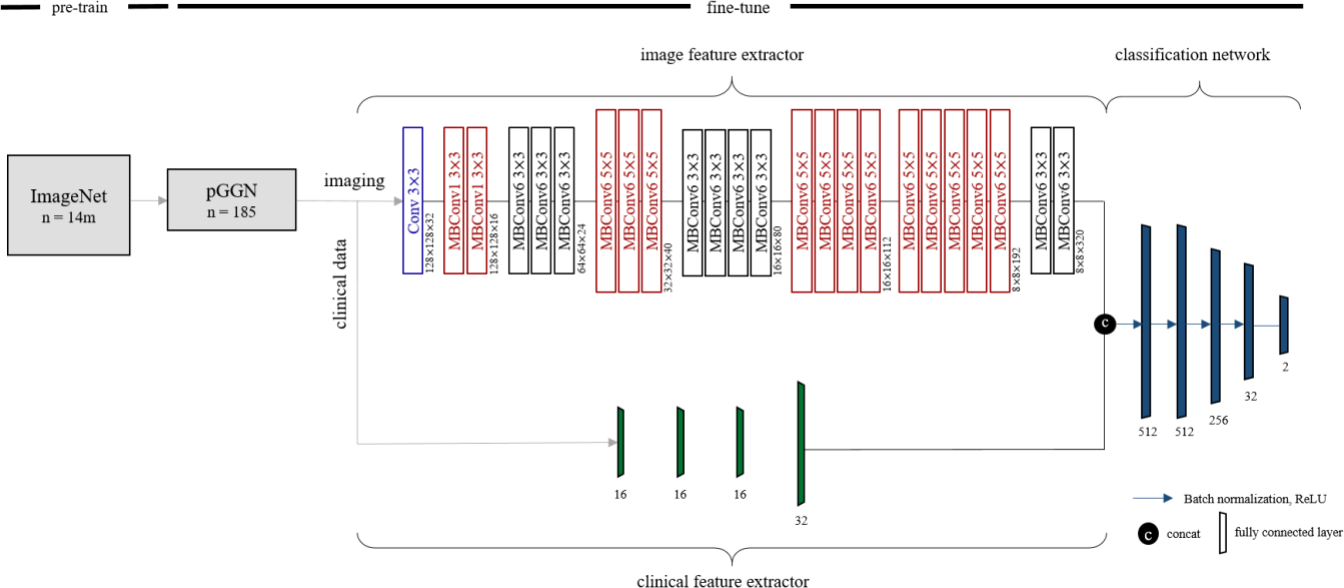
Details of Multimodal EfficientNet-b1 for Lung. Multimodal EfficientNet-b1 for Lung (MENL) consists of an image feature extractor, clinical feature extractor, and classification network. Pre-trained EfficientNet-b1 was used as an image feature extractor. For EfficientNet-b1, MBConv1 and MBConv6 were utilized as basic modules. MBConv1 was composed of depth-wise convolution, SENet (35), and 1×1 convolution. For MBConv6, 1×1 convolution was added before depth-wise convolution of MBConv1.

To predict *EGFR* mutation status in ground-glass opacity lung adenocarcinoma, we designed a deep learning method referred to as Multimodal EfficientNet-b1 for Lung (MENL). This method adopted Efficient as the backbone and is composed of an image feature extractor, clinical feature extractor, and classification network. Based on the mobile inverted bottleneck convolution (MBConv), the EfficientNet varies from b0 to b7 depending on the scaling factor (33, 34). We employed pre-trained EfficientNet-b1 as an image feature extractor. The following clinical factors were fed to the clinical feature extractor for predicting *EGFR* mutation status in pGGN: sex, age, and smoking status. The clinical feature extractor is a separate neural network that consists of four fully connected layers. We concatenated latent variables from the clinical feature extractor to the feature maps from the image feature extractor. Concatenated latent variables were fed into the classification network made of five fully connected layers for predicting *EGF*R mutation status. Finally, *EGFR* mutant probability was obtained by applying softmax to the two nodes in the last layer of the classification network. **Figure 2** shows the details of the MENL.

### Clinical factor model

Clinical factors, sex, age, and smoking status were used as inputs to train a random forest classifier with five decision trees and a maximum depth of 16.

### Radiomics model

Radiomics features were calculated from ROIs. We used Python-based open-source software PyRadiomics (https://pyradiomics.readthedocs.io/) to extract 72 radiomics features in the following four categories: histogram (18 features), shape (14 features), gray-level co-occurrence matrix (GLCM) (24 features), and gray-level size-zone matrix (GLSZM) (16 features) (**Supplementary Table S1**). Additionally, MATLAB-based in-house software was used to calculate five marginal features (Appendix in the **Supporting Information**) (36). In total, we computed 77 radiomics features per ROI. Extracted features were normalized with z-score normalization. The least absolute shrinkage and selection operator (LASSO) was used to select the most useful predictive features for *EGFR* mutation status. Using the selected radiomics features, we applied random forest regression with five decision trees to construct the radiomics model.

### Interpretability of the deep learning model

We utilized gradient-weighted class activation mapping (Grad-CAM) (37) to compute an activation heat map of MENL. Grad-CAM uses gradient information to assign significant values to the feature map to determine where the model focus is when making the prediction. The last CNN layer of MENL was used to create the activation map. After overlaying the activation map and inputting CT image, we analyzed the outcomes of MENL using the four categories of true positive (activation map consistent with positive *EGFR*), true negative, false positive, and false negative cases for model interpretability.

### Training details of the MENL

We used Pytorch (version 1.8.0) for image analysis. Since EfficientNet-b1 has an input image size of 240, we resized the images from 64 to 256. Data augmentation was performed using horizontal and vertical flips with a probability of 0.5. Our MENL was trained on the training set of the primary cohort for 30 epochs. We adopted early-stopping where the model showed the highest accuracy in the technical validation set. Performance was computed on the test set. It took 30 secs to train MENL using the NVIDIA TITAN Xp graphics card.

### Clinical validation

For the independent clinical validation cohort, we included 64 consecutive patients who received an *EGFR*-TKI from January 2010 to December 2015 for stage IV non-small cell lung cancer (NSCLC) and had concurrent GGN(s) that overlapped with a previous study (38). We identified and indexed 83 concurrent GGNs of 64 patients for follow up and grouped these into a response group if concurrent GGN decreased in size or did not change in size, but decreased in density after *EGFR*-TKIs, or as a non-response group if concurrent GGN had an increase in size or density on the last follow-up chest CT (**Figure 1**). We applied MENL to the clinical validation dataset to assess the generalizability and clinical utility of our model and used *EGFR*-TKI treatment response as the reference standard for MENL. That is, the response group was regarded as the *EGFR*-mutant group and the non-response group was regarded as the *EGFR*-wild type group.

### Statistical analysis

To compare clinical variables, ANOVA was conducted for continuous variables and chi-square tests were conducted for categorical variables. To assess the prediction performance of the proposed model, area under the curve (AUC), accuracy, sensitivity, and specificity were calculated to consider both majority and minority classes. All statistical analyses were performed with the statistics tools “scipy,” “statsmodels,” and “sklearn” in Python.

## Results

Among the 185 GGO lung adenocarcinomas, 122 (65.9%) were *EGFR*-mutant and 63 (34.1%) were *EGF*R-wild type. Demographic information and tumor characteristics of the primary cohort are listed in **Table 1**.

**Table 1:** Demographic information and tumor characteristics of the primary cohort (n = 185).

Characteristics	Total (n = 185)	*EGFR*-mutant (n = 122)	*EGFR*-wild type (n = 63)	P-value (*EGFR*-mutant vs. *EGFR*-wild type)
Age (years)^*^	59 (54-64)	58 (54-63)	60 (54-66.5)	0.557
Sex				0.422
Male	75 (40.5)	52 (42.6)	23 (36.5)	
Female	110 (59.5)	70 (57.4)	40 (63.5)	
Smoking history (yes)	66 (35.7)	49 (40.2)	17 (27)	0.076
Operation type				0.317
Lobectomy	69 (37.3)	51 (41.8)	18 (28.6)	
Segmental resection	50 (27)	29 (23.8)	21 (33.3)	
Wedge resection	63 (34.1)	40 (32.8)	23 (36.5)	
Lobectomy + wedge resection	3 (1.6)	2 (1.6)	1 (1.6)	
Pathologic tumor size (mm)^*^	15 (12-19)	15.5 (12-19)	15 (11-19)	0.301
Histopathologic diagnosis				0.552
Minimally invasive adenocarcinoma, T1a(mi)^†^	20 (10.8)	12 (9.8)	8 (12.7)	
Invasive adenocarcinoma, T1a^†^	165 (89.2)	110 (90.2)	55 (87.3)	
Time between CT scan and surgery (days)^*^	4 (1-26)	4 (1-23)	1 (1-27.5)	0.988

Unless otherwise indicated, data are numbers of patients with percentages in parentheses.

^*^Data are median; data in parentheses are interquartile range.

^†^Pathological staging according to the American Joint Committee on Cancer Staging Manual (eighth edition).

EGFR, epidermal growth factor receptor.

### Selected radiomics features for the radiomics prediction model

After feature selection processes, the 11 radiomics features that were selected were as follows: interquartile range, minimum, root mean squared, cluster shade, contrast, maximal correlation coefficient, gray level non-uniformity normalized, elongation, maximum 3D diameter, mean of the cumulative distribution function (CDF) slope, and standard deviation of the (CDF) slope (**Table 2**).

**Table 2:** Selected radiomics features for the radiomics-based prediction model.

First-order	GLCM features	GLSZM feature	Shape features	Margin features
Interquartile Range	Cluster Shade	Gray Level Non-Uniformity Normalized	Elongation	Mean of CDF slope
Minimum	Contrast		Maximum 3D diameter	SD of CDF slope
	Maximal Correlation Coefficient			

GLCM, gray level co-occurrence matrix; GLSZM, gray-level size-zone matrix; CDF, cumulative distribution function; SD, standard deviation.

### Model performance in the test set

**Figure 3:**
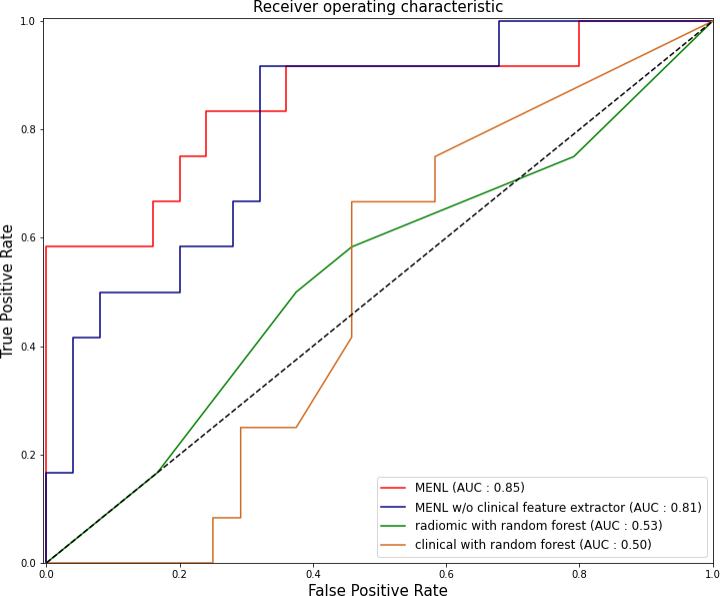
Receiver operating characteristic curves of the Multimodal EfficientNet-b1 for Lung (MENL), MENL without clinical feature extractor, radiomics-based model, and clinical feature-based model in the test set (n = 37) of the primary cohort.

For the test set of the primary cohort (n = 37 of 36 patients), the AUC values of the clinical model (age, sex, and smoking history), radiomics model, and MENL to predict *EGFR* mutations were 0.50, 0.64, and 0.85, respectively (**Table 3** and **Figure 3**).

**Table 3:** Comparison of prediction model performances for the test set of the primary cohort.

Prediction models	AUC	Accuracy	Sensitivity	Specificity
Clinical feature-based model	0.5	0.56	0.25	0.71
Radiomics-based model	0.64	0.56	0.33	0.67
Multimodal EfficientNet-b1 for lung w/o clinical feature extractor	0.81	0.78	0.42	0.96
Multimodal EfficientNet-b1 for lung	0.85	0.81	0.42	1

AUC, area under the curve.

For the test set (n = 37), the median *EGFR*-mutant probability was 0.58 (interquartile range [IQR], 0.57-0.59) in the *EGFR*-mutant group and 0.52 (IQR, 0.48-0.54) in the *EGFR*-wild type group. The discrimination performance of MENL was statistically significant (P < 0.001).

### Ablation study

We added the clinical feature extractor to the existing EfficientNet-b1 to predict *EGFR* mutation status. The newly added clinical feature extractor receives three clinical factors as input and assists in predicting *EGFR* mutation status with CT images. To justify the effectiveness of this design, we deleted the clinical feature extractor in MENL. Thus, the model only consisted of the image feature extractor and classification network. As shown in **Table 3**, the ablation model without the clinical feature extractor showed poorer performance than the MENL.

### Grad-CAM of the deep learning prediction model

**Figure 4:**
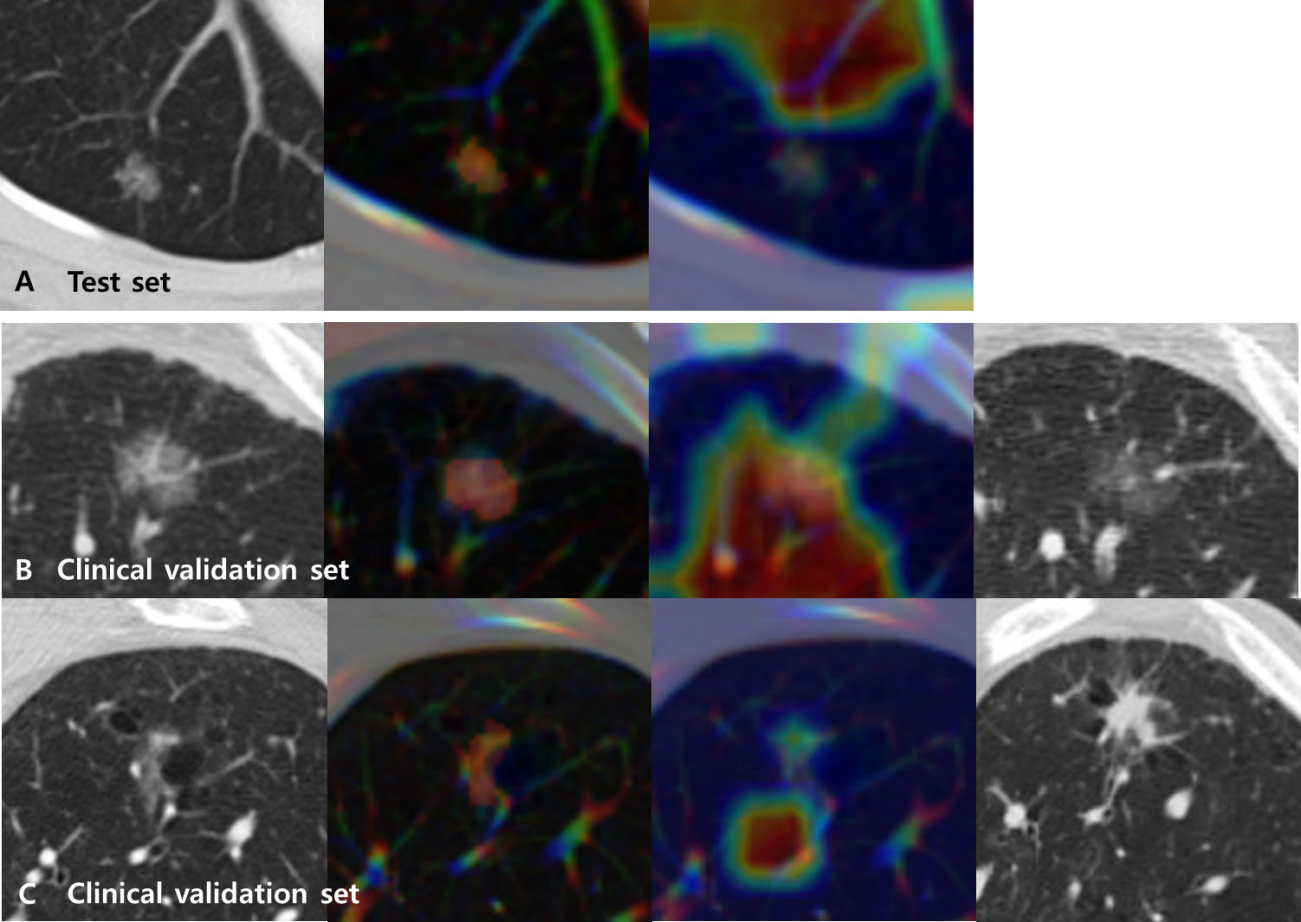
Representative CT images (first from the left) overlaid with regions of interest (ROIs) (second) and Grad-CAMs (third) for Multimodal EfficientNet-b1 for Lung (MENL) interpretation. **(A)** A *EGFR*-mutant correct case (probability 0.62) in the test set. **(B)** A *EGFR*-mutant (response) correct case (probability 0.69) in the clinical validation set. Compared to the baseline CT image (first), the last follow-up CT image after TKI (fourth) demonstrates a decrease in density. **(C)** A *EGFR*-wild type (non-response) correct case (probability 0.51) in the clinical validation set. Compared to the baseline CT image (first), the last follow-up CT image after TKI (fourth) demonstrates an increase in size and density. In all cases, the tumor and its proximal bronchovascular bundle are activated by the MENL.

Grad-CAMs overlaid with CT images for the test set of the primary cohort varied in different tumors. However, a common pattern was that the MENL was highly focused its attention on the proximal bronchovascular bundle of the tumor with tumor inside for *EGFR*-mutant pGGN lung adenocarcinomas. For *EGFR*-wild type pGGN lung adenocarcinomas, a small portion of the tumor and its proximal bronchovascular bundle were activated (**Figure 4** and **Supplementary Figure S1**).

### Clinical validation

The characteristics of the clinical validation cohort are presented in **Supplementary Table S2**. When our MENL was applied to an independent clinical validation set (n = 83 of 64 patients), the AUC was 0.72 (**Table 4**). For the clinical validation set, the median *EGFR*-mutant probability was 0.53 (IQR, 0.50-0.58) in the response group and 0.48 (IQR, 0.48-0.51) in the non-response group. However, discrimination performance was not statistically significant (P = 0.145) (**Table 4**). Grad-CAMs of the MENL in the clinical validation set showed similar patterns to those of the test set in the primary cohort (**Figure 4**).

**Table 4:** Performance of multimodal efficientNet-b1 for lung (MENL) in the clinical validation set.

Data set	AUC	Accuracy	Sensitivity	Specificity	*EGFR*-mutantprobability^*^	P*-*value^†^
Test set (n = 37)	0.85	0.81	0.42	1		
*EGFR*-mutant (n = 25)					0.58 (0.57-0.59)	<0.001
*EGFR*-wild type (n = 12)					0.52 (0.48-0.54)	
Clinical validation set (n = 83)	0.72	0.76	0.6	0.77		
Response (*EGFR*-mutant) (n = 78)					0.53 (0.50-0.58)	0.2168
Non-response (*EGFR*-wild type) (n = 5)					0.49 (0.48-0.52)	

*Data are medians; data in parentheses are interquartile ranges.

†P-values indicate the discrimination performance between the EGFR-mutant and the EGFR-wild type.

MENL, multimodal EfficientNet-b1 for lung; AUC, area under the curve; EGFR, epidermal growth factor receptor.

## Discussion

While tremendous strides have been made in the development of deep learning algorithms in oncology, as evidenced by the surge in publications and published datasets in recent years, there remains a large gap between the evidence for artificial intelligence (AI) performance and evidence for clinical impact (28, 29). There have been no studies demonstrating the clinical utility of deep learning models by applying the model to real-world cancer patients. In this study, we proposed a deep learning model using CT images to predict *EGFR* mutation status among patients with lung adenocarcinoma manifesting as pGGN and demonstrated its clinical utility using an independent cohort made up of patients who received *EGFR*-TKI (83 GGNs of 64 patients) and treatment response as the reference standard. The proposed model showed encouraging results in the primary cohort (AUC = 0.85) and achieved strong performance in the independent clinical validation cohort (AUC = 0.72). Thus, our results are valuable and can be distinguished from previous studies as the first attempt at bridging the AI translational gap between initial model development and routine clinical cancer care, and we demonstrated the clinical feasibility of our MENL model. Our design provides an alternative method to non-invasively assess *EGFR* information and to assist in decision-making when applying TKI as an initial treatment in inoperable or inappropriate situations for surgical treatment of lung adenocarcinoma manifesting as pGGN.

Although there have been studies of deep learning models demonstrating promising performance in assisting lung cancer analysis (24–27), our study is distinguished from prior studies by design and by the relative difficulty of the application. Because we extracted and collected examples of lung adenocarcinoma manifesting as pGGN only according to rigorous criteria not only to construct the primary cohort but also for clinical validation, we engaged in a long-term commitment to gather such patients and their pretreatment CT scans before surgery or TKI. By designing the deep learning model using image feature and clinical feature extractors, we were able to incorporate CT image and clinical factors simultaneously.

The Grad-CAM activation maps convey important regions of cues that dominate the prediction of *EGFR* mutation status. Since deep learning is an end-to-end prediction model that learns abstract mappings between tumor images and *EGFR* mutation status, it is important to explain the prediction process so that users can gain confidence in the prediction process. The activation map focused attention on the proximal bronchovascular bundle of the tumor with the tumor inside. These attention areas were inferred to be strongly related to *EGFR* mutation status by the deep learning model for a lung adenocarcinoma manifesting as a pGGN. Based on our observations, we hypothesized that our deep learning model used information from the proximal bronchovascular bundle of the tumor to make predictions.

In this study, the radiomics model achieved an AUC of 0.64, which was inferior to those of existing radiomics studies predicting *EGFR* mutation for lung adenocarcinoma (16–18). This shortcoming could be due to unique characteristics of pGGN such as extremely homogenous and negative CT density causing skewness of data, which makes it difficult to discriminate by morphology or radiomics. Thus, we believe deep learning methods can overcome such limitations in imaging prediction for specific subjects such as pGGN adenocarcinomas. In addition, previous studies used clinical factors to predict *EGFR* mutation status. For example, clinical factors such as age, sex, smoking status, tumor stage, and predominant subtype were used to build prediction models for *EGFR* mutation status. These studies achieved AUC ranging from 0.68-0.84 in different populations (39–41). In contrast, our clinical model (age, sex, and smoking history) was subpar with an AUC value of 0.5 (**Table 3**). One reason for this poor discrimination performance could be that our study subjects consisted of all pGGN adenocarcinomas. However, when the clinical model was added to the deep learning model, prediction performance improved from AUC 0.81 to AUC 0.85. Thus, clinical features (young age, female, and non-smoker) traditionally considered to be significant factors for predicting *EGFR* in lung adenocarcinoma retain important roles in pGGN adenocarcinoma.

In this study, our deep learning model demonstrated advantages since it can mine abstract features that are difficult to extract with conventional methods but are important for identifying *EGFR* mutation status. Compared with previously reported hand-crafted semantic or radiomics features, the deep learning model has additional advantages. First, the deep learning model extracts multi-level features from low-level visual characteristics to abstract features that are directly related to *EGFR* information through a hierarchical neural network structure. Second, the deep learning model does not require time-consuming tumor boundary annotation, which is a major advantage over the radiomics approach. Moreover, the microenvironment of tumors and the relationships between tumors and surrounding tissues such as lung parenchyma and bronchovascular structure are inherently considered in the deep learning model because the peripheral regions are typically included in the rectangular image patch. Third, the deep learning model is fast and easy to use, requires only the CT image as input, and predicts *EGFR* mutation status directly without further human input.

Despite the encouraging performance of our deep learning model, this study has several limitations. First, we only examined patients in an East Asian population. However, *EGFR* mutation rate can be affected by regional variation in humans. In future work, samples from multiple areas of the world will be necessary to test whether the deep learning model can be generalized to other populations. Second, although the deep learning model shows better performance than models using clinical features and radiomics, how to optimally combine these two models remains an open question. The predictive performance of our model may be improved if we adopt other advanced approaches to combine these two models. Third, our predictive model could not be applied for the predictive model with solid and solid dominant nodules for investigation of its scalability. In this study we focused on only pure GGNs because we were concerned with increasing cases of multiple pGGNs on screening chest CT and their early management strategy. However, application of our predictive model for solid and solid dominant nodules could be valuable and helpful especially on unresectable cases. Thus, we plan to expand cases with solid and solid dominant nodules and devise another cohort to validate our results for the next study. Finally, the number of patients in the clinical validation set was small and there were only five *EGFR*-wild type cases which made insufficient balance between ‘Response (EGFR-mutant) group (n = 78)’ and ‘Non-response (EGFR-wild type) group (n = 5)’. This might have limited the statistical power for validating the performances of the prediction models. Nevertheless, a total of 83 subjects could be meaningful because it is difficult to find such number of cases that have relatively long-term, serial follow-up CT scans. This data imbalance could be resolved in future work by using a larger number of non-response (EGFR-wild type) cases.

In conclusion, our preoperative CT-based deep learning model was able to predict *EGFR* mutations in patients with lung adenocarcinomas manifesting as pGGN. Our deep learning model outperformed the radiomics model in the detection of *EGFR* mutations. The combination of deep learning and clinical models showed further performance improvements in *EGFR* prediction and demonstrated its clinical utility in the real-world population. This deep learning model provides a non-invasive method to predict *EGFR* mutation status, can be used easily in routine CT diagnosis, and may facilitate clinical decision-making in the era of precision medicine.

## Data availability statement

The original contributions presented in the study are included in the article/**Supplementary Material**. Further inquiries can be directed to the corresponding authors.

## Ethics statement

The studies involving human participants were reviewed and approved by Institutional Review Board of the Samsung Medical Center. Written informed consent for participation was not required for this study in accordance with the national legislation and the institutional requirements. Written informed consent was not obtained from the individual(s) for the publication of any potentially identifiable images or data included in this article.

## Author contributions

HYL and HP takes full responsibility for the content of the manuscript, including data and analysis. HJY, JC, and HYL contributed to the study concept, design, interpretation and writing. EK, S-WU, NK, WK, and GK contributed to acquisition of data or analysis.

## Funding

This work was supported by the National Research Foundation of Korea (NRF) grant funded by the Korea government (MSIT) (No.NRF-2022R1A2C1003999), the Future Medicine 20*30 Project of the Samsung Medical Center [#SMX1210781], the Institute for Basic Science (IBS-R015-D1), the National Research Foundation of Korea (NRF-2020M3E5D2A01084892), the Ministry of Science and ICT of Korea under the Information Technology Research Center program (IITP-2020-2018-0-01798), the AI Graduate School Program (2019-0-00421), the ICT Creative Consilience program (IITP-2020-0-01821), and the Artificial Intelligence Innovation Hub program (2021-0-02068).

## Conflict of interest

The authors declare that the research was conducted in the absence of any commercial or financial relationships that could be construed as a potential conflict of interest.

## Publisher’s note

All claims expressed in this article are solely those of the authors and do not necessarily represent those of their affiliated organizations, or those of the publisher, the editors and the reviewers. Any product that may be evaluated in this article, or claim that may be made by its manufacturer, is not guaranteed or endorsed by the publisher.
