# Supplementary material for: Deep learning analysis to predict EGFR mutation status in lung adenocarcinoma manifesting as pure ground-glass opacity nodules on CT
Source: Front Oncol. 2022 Sep 2;12:951575. doi: 10.3389/fonc.2022.951575 (PMC9478848; doi:10.3389/fonc.2022.951575)

**Appendix**

Intensity changes are important to define tumors. Based on this knowledge, the margin feature is computed through the slope of the cumulative distribution function (CDF) computed from the intensity histogram within the tumor region. The margin features can distinguish between well-defined and ill-defined tumors well (1). We utilized mean, standard deviation (SD), 75th percentile, skewness, and kurtosis of the CDF slope as margin features.

1. Cho HH, Lee G, Lee HY, Park H. Marginal radiomics features as imaging biomarkers for pathological invasion in lung adenocarcinoma. *Eur Radiol* (2020) 30(5):2984-94. doi: 10.1007/s00330-019-06581-2

**Table S1 |** Radiomic features used to predict EGFR mutation status.

| **First-order features** | **GLCM features** | **GLSZM features** | **Shape features** | **Margin features** |
| --- | --- | --- | --- | --- |
| 10th percentile | Autocorrelation | Gray Level Non-Uniformity | Elongation | Mean of CDF slope |
| 90th percentile | Cluster Prominence | Gray Level Non-Uniformity Normalized | Flatness | SD of CDF slope |
| Energy | Cluster Shade | Gray Level Variance | Least Axis Length | 75th percentile of CDF slope |
| Entropy | Cluster Tendency | High Gray Level Zone Emphasis | Major Axis Length | skewness of CDF slope |
| Interquartile Range | Contrast | Large Area Emphasis | Maximum 2D diameter (Column) | kurtosis of CDF slope |
| Kurtosis | Correlation | Large Area High Gray Level Emphasis | Maximum 2D diameter (Row) |  |
| Maximum | Difference Average | Large Area Low Gray Level Emphasis | Maximum 2D diameter (Slice) |  |
| Mean Absolute Deviation | Difference Entropy | Low Gray Level Zone Emphasis | Maximum 3D diameter |  |
| Mean | Difference Variance | Size-Zone Non-Uniformity | Mesh Volume |  |
| Median | Inverse Difference | Size-Zone Non-Uniformity Normalized | Minor Axis Length |  |
| Minimum | Inverse Difference Moment | Small Area Emphasis | Sphericity |  |
| Range | Inverse Difference Moment Normalized | Small Area High Gray Level Emphasis | Surface Area |  |
| Robust Mean Absolute Deviation | Inverse Difference Normalized | Small Area Low Gray Level Emphasis | Surface Area to Volume ratio |  |
| Root Mean Squared | Informational Measure of Correlation 1 | Zone Entropy | Voxel Volume |  |
| Skewness | Informational Measure of Correlation 2 | Zone Percentage |  |  |
| Total Energy | Inverse Variance | Zone Variance |  |  |
| Uniformity | Joint Average |  |  |  |
| Variance | Joint Energy |  |  |  |
|  | Joint Entropy |  |  |  |
|  | Maximal Correlation Coefficient |  |  |  |
|  | Maximum Probability |  |  |  |
|  | Sum Average |  |  |  |
|  | Sum Entropy |  |  |  |
|  | Sum of Squares |  |  |  |

*GLCM, gray level co-occurrence matrix; GLSZM, gray-level size-zone matrix; CDF, cumulative distribution function; SD, standard deviation.*

**Table S2 |** Characteristics of the 83 concurrent pure ground-glass opacity nodule of clinical validation cohort (*n* = 64).

| **Characteristics** | **Total (n = 83)** |
| --- | --- |
| Age (years)^*^ | 57 (53-63) |
| Sex |  |
| Male | 30 (36.1) |
| Female | 53 (63.9) |
| Smoking history (yes) | 23 (27.7) |
| Size of pGGN (mm)^*^ | 7.1 (6.0-9.7) |
| Type of initial *EGFR*-TKI |  |
| Gefitinib | 59 (71.1) |
| Erlotinib | 22 (26.5) |
| Erlotinib → Gefitinib | 2 (2.4) |
| Best response of *EGFR*-TKI for concurrent pGGN |  |
| Partial remission or stable disease (designated as response group) | 78 (94) |
| Progressive disease (designated as non-response group) | 5 (6) |
| From concurrent pGGN detection to initiation of *EGFR*-TKI (days)^*^ | 336 (191-520) |
| Duration of *EGFR*-TKI (days)^*^ | 312 (84-623) |
| From *EGFR*-TKI discontinuation to last follow-up (days)^*^ | 298 (67-662) |

*Unless otherwise indicated, data are the number of patients with percentages in parentheses.*

**Data are median; data in parentheses are interquartile range.*

*EGFR, epidermal growth factor receptor; TKI, tyrosine kinase inhibitor; pGGN, pure ground-glass opacity nodule.*

**Figure S1 |** Additional representative CT images (left) overlaid with regions of interest (ROIs) (middle) and Grad-CAMs (right) for the Multimodal EfficientNet-b1 for Lung (MENL). (A) A *EGFR*-mutant correct case (probability 0.63) in test set. (B) A *EGFR*-wild type correct case (probability 0.47) in test set.


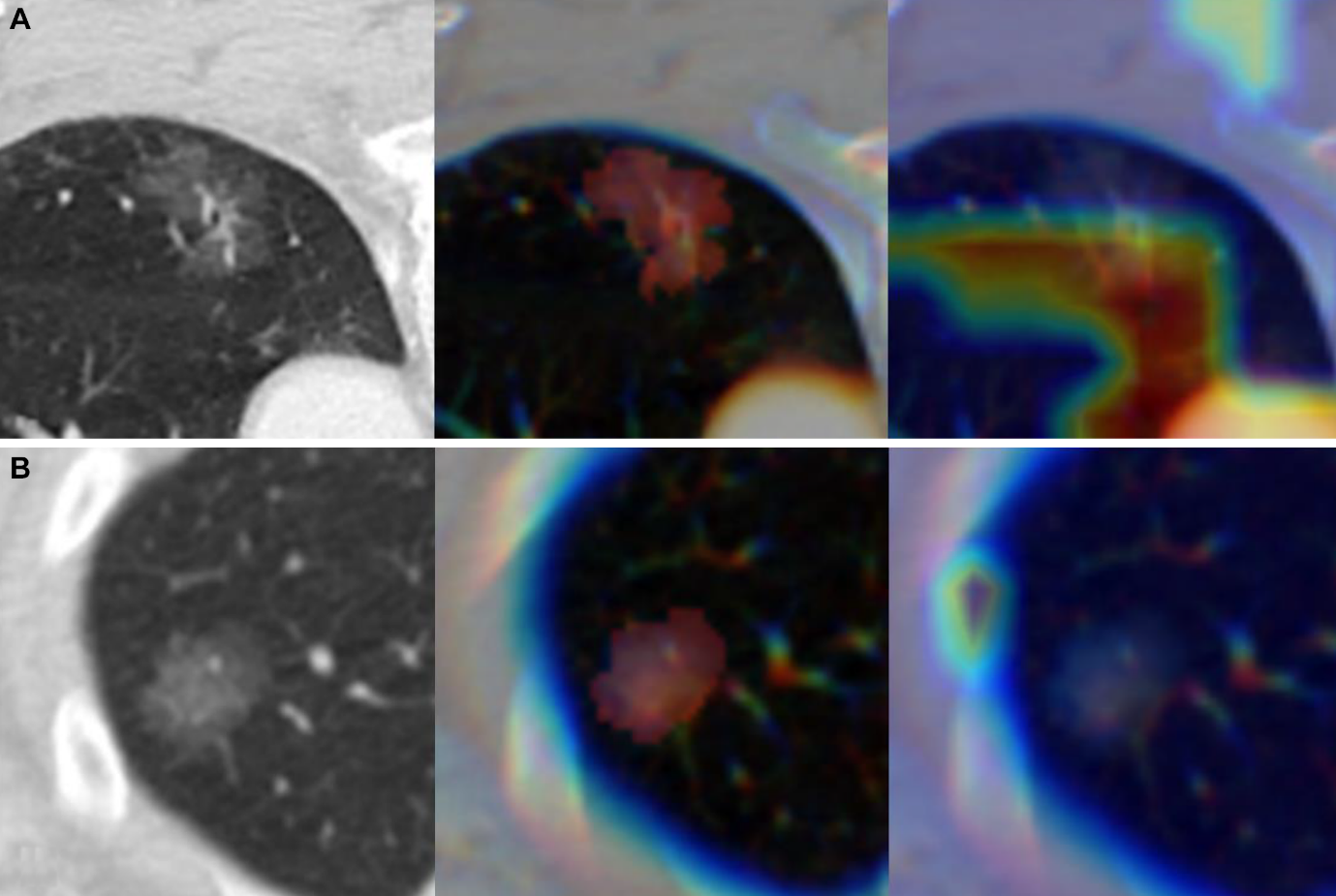

Supplement: Supplementary file 1 [file DataSheet_1.docx]
